# Impact of an integrated health, nutrition, and early child stimulation and responsive care intervention package delivered to preterm or term small for gestational age babies during infancy on growth and neurodevelopment: study protocol of an individually randomized controlled trial in India (Small Babies Trial)

**DOI:** 10.1186/s13063-024-07942-z

**Published:** 2024-02-08

**Authors:** Ranadip Chowdhury, Rukman Manapurath, Ingvild Fossgard Sandøy, Ravi Prakash Upadhyay, Neeta Dhabhai, Saijuddin Shaikh, Harish Chellani, Tarun Shankar Choudhary, Abhinav Jain, Jose Martines, Nita Bhandari, Tor A. Strand, Sunita Taneja, on behalf of the Small Babies Trial Group

**Affiliations:** 1 Society for Applied Studies, 45 Kalu Sarai, New Delhi, India; 2 Centre for International Health, University of Bergen, Bergen, Norway; 3 Centre for Intervention Science in Maternal and Child Health, Department of Global Public Health and Primary Care, University of Bergen, Bergen, Norway; 4 Hamdard Institute of Medical Sciences & Research, New Delhi, India; 5 Department of Research, Innlandet Hospital Trust, Brumunddal, Norway

**Keywords:** Small vulnerable newborns, Intrauterine growth restriction, Growth failure, Child health, Early child stimulation, Responsive stimulation, preterm, Small for gestational age

## Abstract

**Background:**

Preterm and term small for gestational age (SGA) babies are at high risk of experiencing malnutrition and impaired neurodevelopment. Standalone interventions have modest and sometimes inconsistent effects on growth and neurodevelopment in these babies. For greater impact, intervention may be needed in multiple domains—health, nutrition, and psychosocial care and support. Therefore, the combined effects of an integrated intervention package for preterm and term SGA on growth and neurodevelopment are worth investigating.

**Methods:**

An individually randomized controlled trial is being conducted in urban and peri-urban low to middle-socioeconomic neighborhoods in South Delhi, India. Infants are randomized (1:1) into two strata of 1300 preterm and 1300 term SGA infants each to receive the intervention package or routine care. Infants will be followed until 12 months of age. Outcome data will be collected by an independent outcome ascertainment team at infant ages 1, 3, 6, 9, and 12 months and at 2, 6, and 12 months after delivery for mothers.

**Discussion:**

The findings of this study will indicate whether providing an intervention that addresses factors known to limit growth and neurodevelopment can offer substantial benefits to preterm or term SGA infants. The results from this study will increase our understanding of growth and development and guide the design of public health programs in low- and middle-income settings for vulnerable infants.

**Trial registration:**

The trial has been registered prospectively in Clinical Trial Registry – India # CTRI/2021/11/037881, Registered on 08 November 2021.

**Supplementary Information:**

The online version contains supplementary material available at 10.1186/s13063-024-07942-z.

## Administrative information

**Table:** 

Title {1}	Impact of an integrated health, nutrition, and early child stimulation and responsive care intervention package delivered to preterm or term small for gestational age babies during infancy on growth and neurodevelopment: study protocol of an individually randomized controlled trial in India (Small Babies Trial)
Trial registration {2a and 2b}.	The trial has been registered prospectively in Clinical Trial Registry – India # CTRI/2021/11/037881, Registered on 08 November 2021. The items of WHO Trial Registration Dataset are available in the Additional file Table 6.
Protocol version {3}	Date 24.07.2023 and version 3.0.
Funding {4}	Centre for Intervention Science in Maternal and Child Health (CISMAC) at the University of Bergen (Bergen, Norway)
Author details {5a}	Ranadip Chowdhury^1^, Rukman Manapurath^1,2^, Ingvild Fossgard Sandøy^2,3^, Ravi Prakash Upadhyay^1^, Neeta Dhabhai^1^, Saijuddin Shaikh^1^, Harish Chellani^1^, Tarun Shankar Choudhary^1^,^3^, Abhinav Jain^4^, Jose Martines^3^, Nita Bhandari^1^, Tor A Strand5, Sunita Taneja^1^ on behalf of the Small Babies Trial Group ^1^Society for Applied Studies, New Delhi, India ^2^Centre for International Health, University of Bergen, Bergen, Norway ^3^Centre for Intervention Science in Maternal and Child Health, Department of Global Public Health and Primary Care, University of Bergen, Bergen, Norway ^4^Hamdard Institute of Medical Sciences & Research, New Delhi, India ^5^ Department of Research, Innlandet Hospital Trust, Brumunddal, Norway
Name and contact information for the trial sponsor {5b}	Sunita Taneja Society for Applied Studies sunita.taneja@sas.org.in
Role of sponsor and funder {5c,5d}	The sponsor controls the design, analysis, interpretation, and reporting of the trial. The funder does not have a role in the design of the study, its execution, analyses, interpretation of the data, or decision to submit results.

## Introduction

### Background and rationale {6a}

Preterm births (born before 37 completed weeks of gestation) and babies being term small for gestational age (term SGA—birth weight for gestational age below the 10th percentile) present significant global health problems [1, 2]. It is estimated that globally, 11.9 million live births (8.8% of all live births) are preterm, while approximately 21.9 million (16.3%) neonates are born term SGA [3]. The proportion of babies born preterm and term SGA is higher in South Asia compared to other parts of the world [1, 2, 4], and within South Asia, India has the highest proportion of preterm (13.6%) and term SGA (36.5%) babies [1, 2]. These babies comprise 40% of all live births and 65–70% of infants with underweight, stunting, and wasting in Delhi, India [5].

Babies born preterm and term-SGA are vulnerable to serious infections and feeding difficulties [6]. This contributes to an increased risk of death, growth impairment, and neurodevelopmental disorders in early and later life [7, 8]. Evidence from low- and middle-income countries (LMICs) indicates that preterm and term SGA babies have 2–3 times increased risk of being underweight at age 12 to 60 months compared to term appropriate for gestational age (AGA) babies [9]. A similar magnitude of risk was also observed for stunting and wasting [9]. Evidence from systematic reviews suggest that children born as term SGA babies have 0.3 to 0.5 SD lower neurodevelopment scores between 1 and 12 years of age compared to those born term AGA [8, 10, 11]. Preterm and term SGA babies are also at increased risk of neurodevelopment impairment. Preterm babies have lower cognitive and fine motor skills scores (SMD: − 0.70; 95% CI: − 0.73 to − 0.66) and an increased risk of behavioral problems at school age compared to term babies. Additionally, preterm babies have a higher likelihood of motor skill impairment with 40% experiencing mild impairment and 20% experiencing moderate impairment [12, 13]. Preterm and term SGA babies may additionally exhibit a more difficult and less predictable temperament than term AGAs that can pose a challenge for caregivers to provide appropriate responsive care, possibly aggravating the delayed neurodevelopment [14–17].

The factors influencing growth and neurodevelopment in preterm and term SGA infants are multifactorial. It encompasses a range of factors including infant morbidities, breast feeding practices, maternal and infant nutrition, and maternal psychosocial status. Research indicates that standalone interventions targeting these factors have modest and sometimes inconsistent effects on growth and neurodevelopment [18–29]. Health interventions such as prevention of disease through sanitation, treatment of diarrhea, and immunization were found to increase linear growth among children in developing countries [30–32]. Enteral iron supplementation has been found to improve length among preterm and LBW infants [MD 0.69 cm, 95% CI 0.01 to 1.37] in the first 6 months of life [33, 34], and kangaroo mother care (KMC), which includes exclusive breastfeeding, has been shown to improve weight [4.08 g/day (2.30 to 5.86)] and length [0.21 cm/week, (0.03 to 0.38)] in low birth weight infants [21, 34, 35]. Early child stimulation activities among preterm infants have been found to improve cognitive (developmental quotient (DQ): SMD: 0.32 SD; 95% CI: 0.16 to 0.47) and motor outcomes in infancy (motor scale DQ: SMD 0.10 SD, 95% CI: 0.01 to 0.19) [27]. For greater impact, intervention may be needed in several domains, i.e., health, nutrition, and psychosocial care and support, delivered concurrently [36, 37]. Improving the mother’s postnatal health, nutritional status, and psychological well-being may also be critical for implementing interventions promoting optimal growth and neurodevelopment of preterm and term SGA babies [37, 38].

The first 1000 days of life are crucial for subsequent growth and brain development. Insufficient nutrition, repeated infections, and sub-optimal care have negative impacts on both [39, 40]. This is particularly important for preterm and term SGA infants, as achieving postnatal catch-up growth within the first 6 months of life is strongly associated with better neurodevelopment in preschool, school-age, and at later stages [36, 41–44]. Studies have shown that the likelihood of catch-up growth in preterm babies from LMICs is limited [45]. There is very limited evidence regarding catch-up growth in term SGA infants in LMICs.

Identification of an intervention package that substantially improves the growth and neurodevelopment of preterm and SGA infants may hold the potential to reduce undernutrition in infancy and may contribute to improved health, educational achievement, and economic status in adulthood for a substantial proportion of the Indian population [46]. This package should include intervention that have been documented to have at least modest effects when delivered on their own and are expected to achieve synergistic effects on growth and neurodevelopment when combined. We are conducting an individually randomized controlled trial in low to middle socioeconomic neighborhoods of Delhi to ascertain the efficacy on growth and neurodevelopment of an integrated package of health, nutrition, and early child stimulation and responsive care intervention delivered concurrently to term SGA and preterm infants and their mothers. The comparator group in this study are infants who are receiving routine care from either government or private hospitals. This comparison is crucial to determine whether the intervention provides any additional benefits over and above the current standard of care.

### Objectives {7}

The primary objectives are to estimate the efficacy of concurrent delivery of a health, nutrition, and early child stimulation and responsive care intervention package during the first year of life on attained weight and weight for age *z* score (WAZ) at 12 months of age for (i) preterm infants and their mothers and (ii) term SGA infants and their mothers.

The secondary objectives are to determine the effects of the same package on mortality, morbidity, nutritional status, and neurodevelopment in infants and nutritional status and depressive symptoms among mothers in the first year of infant age. We will also estimate the effect on household consumption and expenditures at 6 and 12 months of infant age.

### Trial design {8}

An individually randomized controlled trial with parallel design (1:1), stratified by preterm and term small for gestational age (SGA). The trial hypothesis is that the integrated and concurrent delivery of interventions in the domain of health, nutrition, and early child stimulation and responsive care to preterm or term SGA infants and their mothers during infancy will increase their mean attained weight and weight-for-age *z* scores by at least 0.2SD or more at 12 months of age. Blinding will be implemented at the outcome assessor and data analyst level to minimize bias. All design elements of the trial have been carefully considered to ensure the validity and reliability of the trial results.

## Methods: participants, interventions, and outcomes

### Study setting **{9}**

The study is an individually randomized trial and is being conducted in urban and peri-urban low to middle socioeconomic neighborhoods of South Delhi, India [47]. In this setting, the proportions of infants born preterm (~ 15%) and SGA (~37%) are similar to the national average [5]. The prevalence of stunting and underweight at 12 months of age in preterm infants is 31% and 20% and in term SGA 34% and 36%, respectively [48]. Approximately 45% of women have secondary level or higher education. Almost all (95%) births are institutional, with 80% taking place in public health facilities. Four fifths (around 80%) of mothers return home within 72 h of delivery.

### Eligibility criteria **{10}**

#### Inclusion criteria

Preterm or term SGA infants and their mothers with ultrasound dating scan done within 20 weeks of gestation are eligible for participation.

#### Exclusion criteria

Infants with congenital malformations (affecting feeding or the ability to take measurements), mothers intending to leave the study area within the next 12 months, or babies or mothers who are hospitalized for more than 14 days post-delivery are excluded from the study.

### Sample size {14}

There are two strata: preterm and term SGA. Sample sizes are calculated for 90% power and 95% confidence for each stratum. Assuming a minimum of 0.20 SD mean difference in weight (which translates to a 200 g weight difference) or WAZ scores between the intervention and control arms at 12 months, a total of 1054 infants per strata, i.e., (527 infants per arm per strata) are required [5].

### Recruitment {15}

Accounting for a possible 20% loss to follow-up from enrolment to 12 months of age, we will need 650 infants per arm per stratum. We will therefore enroll a total of 2600 infants (1300 preterm and 1300 term SGA).

With 650 infants in each stratum in each arm, assuming a prevalence of underweight of 25% in the control arm at 12 months of age, we will be able to detect a 30% relative reduction in underweight, with 90% power and 95% confidence level. A narrow effect size in the hypothesis has been considered to adjust for some earlier impact of similar intervention in the study area, by our team [1]. We will also be able to detect a difference of 3 composite standard score–points (0.2 SD) in the domains of the Bayley Scales of Infant and Toddler Development, 3rd Edition (BSID-III).

Assuming 25% of babies are term SGA and 15% preterm, and 40% attrition from pregnancy identification to enrolment, we will probably need gestational age assessment through dating ultrasounds in ~13,000 pregnancies [1]. The sample size estimation for this study was conducted using *power* commands in STATA.

### Intervention description {11a}

The intervention has three domains—health, nutrition, and early child stimulation-and-responsive care—and is being delivered from birth until 12 months of age. These were selected based on their impact on growth and developmental outcomes in preterm, term SGA, and LBW infants (Table 1). The details of this intervention are listed in Additional file 1.

**Table 1:** Summary of the intervention

Component	Intervention	Control
**Infants**		Care routinely sought from usual sources -government (free of cost) and private providers
Health	Illness ascertainment through reports by caregivers and by team, at home visits Facilitation of medical care-seeking/access Provision of zinc and ORS for the management of diarrhea Counselling on immunization and handwashing practices	
Nutrition	*0 to 6 months* Growth monitoring based on WHO standard growth charts. Assessment by physician, lactation counsellor, and psychologist (for mothers) if there is growth failure Management of severe acute malnutrition (SAM) Counselling and support for exclusive breastfeeding Expressed breastmilk feeding, if indicated Kangaroo mother care during the neonatal period Lactational counselling and additional breastfeeding support for breastfeeding problems Micronutrient supplementation of iron, vitamin D, zinc, or other B vitamins *7 to 12 months* Counselling on continued breastfeeding, complementary feeding, responsive feeding, food hygiene, and immunization Iron and folic acid supplementation	
Early child development	Counselling and demonstration of early child play and responsive care for the infant	
**Mother**		
Health	Counselling on postnatal check-ups and family planning	
Nutrition	*6 months postpartum* Nutritional supplementation to mother in the form of healthy snacks, calcium and vitamin D, iron and folic acid supplementation, and multiple micronutrient tablets	
Psychosocial support	Promotion of positive thinking and problem-solving skills	

In the health domain, the key components are prevention, early identification, and management of infections [49]. Mothers are counselled to seek vaccination of the infants according to the national immunization schedules. They are also counselled and given demonstrations on correct handwashing practices during food preparation and feeding of the baby. Any illnesses reported by caregivers are managed at the study clinic in the collaborating hospital or any nearby health facilities [50, 51].

From enrollment to 6 months, mothers are counselled and supported to breastfeed their infants exclusively. For late preterm infants (34 to 37 weeks) who show signs of tiring quickly and suckle for less than 5 min, the mother is encouraged to give expressed breast milk feeding after each breastfeed. For infants less than 34 weeks gestation, expressed breastmilk is given following each direct feed. Mothers are taught to express, store and feed breastmilk [52, 53].

Mothers are taught to keep the baby in the skin-to-skin contact (SSC) position, i.e., upright between her breasts, and counselled to give SSC as long as possible during the day and night, in a semi-reclining or supine position, till the baby is 28 days old or wriggles out.

All infants are provided micronutrient supplementation daily as per 2022 WHO guidelines for preterm or LBW infants (iron 2–4 mg/kg/day, vitamin D 800 IU/day, and zinc 2–3 mg/kg/day) [54].

Caregivers of infants aged 6 to 12 months are counselled on timely introduction of complementary foods at 6 months, on the frequency of feeding and types of food to be fed and their amounts, and recipes for energy and nutrient-dense meals made from locally available and culturally acceptable foods are shared. A daily milk cereal mix packet (125 Kcal per day, and 5 g protein including 80% to 100% recommended daily allowances (RDA) of micronutrients) is provided to all infants (see Additional file 2 for detailed composition). The nutritional supplement provides around 50 to 60% of the daily energy requirement between 6 to 12 months of age, assuming the infant is breastfed [55]. Mothers are counselled and supported to continue breastfeeding till at least 12 months of age.

Growth is monitored fortnightly in the first 2 months of life and subsequently monthly to identify growth failure. Growth failure is defined using both attained growth and growth velocity. Attained growth is calculated based on WAZ and LAZ in the first 6 months and WLZ and LAZ in the next 6 months using WHO growth standards. Growth velocity is monitored based on WHO weight velocity standards [56]. Weight velocity below the 15th centile for term SGA and below the 25th centile for preterm infants is defined as growth failure [5]. Attained growth and growth velocity of infants in the intervention arm are monitored using an electronic monitoring system, which calculates growth indices (attained and velocity *z* scores) in real-time.

Infants with growth failure are referred to the study clinic and assessed for morbidity, breastfeeding, and complementary feeding practices. In addition to support on how to manage the morbidity of their infants, mothers of infants under 6 months with growth failure are provided with additional counselling on the importance of exclusive breastfeeding. Infants older than 6 months with growth failure are given one packet of additional food supplement (125 Kcal, ~ 5 g protein) per day, in addition to breastfeeding support.

The intervention for early child development (ECD) have been adapted from the “Care for Child Development” manual developed by WHO and UNICEF [57]. The strategies involve fostering a strong connection between the research team and the mother or primary caregiver, aiming to boost her motivation for better childcare. It includes observing the mother’s play and interaction with the child and providing recommendations for and demonstrations of age-appropriate activities for her to engage with her child. Additionally, assistance is being provided to the mother during the activities, while encouraging her to practice them independently. The developmental screening tool, Ages and Stages Questionnaire version III (ASQ-3) will be administered at 4, 6, 9, and 11 months of infant age to identify early delays in child development [58]. Infants whose parents have concerns about developmental delay and those with ASQ-3 scores below the age-specific cut-off are assessed by the study psychologist and referred to a developmental pediatrician if needed.

Mothers receive counselling on postnatal check-ups and family planning practices. Contraceptives are provided if requested. Mothers are provided with ~1 RDA of daily multiple micronutrient supplements (Additional file 3 for the detailed composition of the micronutrient supplement, Riconia Silver) and locally prepared snacks that align with the Indian Council of Medical Research (ICMR) guidelines for 6 months post-partum [59]. The daily snacks provide 600 kcal with 25–30% of energy (150–180 kcal) from fats and 13% of energy from proteins (80 kcal). It contains 20 g of protein from a mix of plant- and animal sources, with ∼30% (5.4–6 g) of the protein coming from a dairy source.

All mothers are also counselled for their psychological well-being using a module adapted from the WHO Thinking Healthy Manual [60]. The intent is to promote psychological well-being and strengthen the mother’s problem-solving skills. The adapted version of the module emphasizes five basic principles—empathetic listening, guided discovery using pictures, family engagement, problem-solving, and behavioral activation—and is aligned to the local context in order to make it easier for the mothers to comprehend and practice. Patient Health Questionnaire 2-item (PHQ-2) along with the assessment of suicidal ideation are being used to screen for postpartum depression. Mothers suspected of having depression are referred to a clinical psychologist for further evaluation.

### Comparison arm {6b}

The children in the comparison arm receive routine home visits by government health staff [61].

All participants (women and infants) in both arms are free to access their usual care pathways including free services provided through the government health system.

### Outcome measures {12}

The primary outcomes include attained weight, and weight for age *Z* scores at 12 months of age.

The secondary outcomes along with timing of their measurements are listed in Additional file 4. The key secondary outcomes for children are proportion stunted, wasted, and underweight at 6 and 12 months; overweight or obesity at 12 months; weight and length velocities between birth to 6 months and 6 to 12 months; neurodevelopment (composite cognitive, language, motor, socio-emotional, temperament and HOME scores; Mean Global Scale for Early Development score) at 12 months [62]; morbidity and hospitalization from birth to 12 months; and dietary assessment, micronutrients, and anemia status at 12 months (in a sub-sample).

The key secondary outcomes for mothers (Additional file 5) are depressive symptoms, nutritional status during the postpartum period, and household consumption and expenditures at 6 and 12 months of infant age, as well as dietary assessment at 3 and 6 months (in a sub-sample).

## Study procedures

### Surveillance, follow-up, screening, and enrollment

**Fig. 1:**
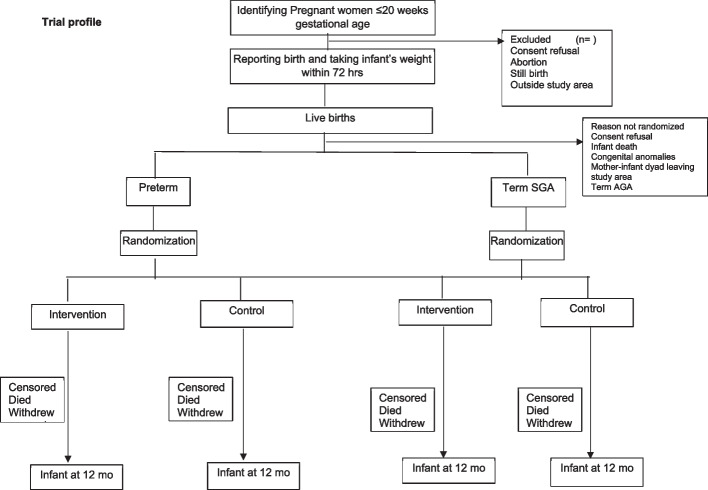
Trial profile

A pregnancy surveillance team (PST) conducts a door-to-door survey to list all pregnancies within 20 weeks of gestation based on the last menstrual period (LMP) or previous ultrasound and takes consent for ultrasound, regular contacts during pregnancy, and birth weight measurement within 72 h of childbirth. The team offers to facilitate the dating ultrasound in designated USG centers and transportation for the same. The details of pregnant women with gestational age < 20 weeks based on ultrasound are communicated to the pregnancy follow-up, screening, and enrolment team (PSE) (Fig. 1).

During follow-up, pregnant women are counselled by field assistants (FAs) to register and deliver in a hospital, attend regular antenatal care clinics, consume an adequate diet, recognize danger signs in pregnancy, and initiate of breastfeeding early after birth. The field assistants periodically contact the identified women over the phone (through home visits if phone calls are unsuccessful), more frequently in the last trimester. For all live births, the team measures the baby’s weight within 72 h of birth and screens using inclusion and exclusion criteria. If the inclusion criteria are met, there are no exclusion criteria, and consent is given; the mother-infant pair is randomized to the intervention or control arm in the relevant stratum (preterm or term SGA). Post enrollment, baseline information is collected, including sociodemographic characteristics and recent childbirth related details including care received after delivery.

### Randomization, allocation concealment, and masking {16a, 16b, 16c, 17a}

The randomization list was prepared by an independent statistician using random permuted blocks of varying sizes, stratified by preterm and term SGA infants. The arm allocation is done using a web-based system at the time of enrolment. There are no additional criteria for discontinuation or modification of allocated intervention. Masking of the study teams is not possible because of the nature of the intervention. However, attempts are made to keep the independent outcome ascertainment team unaware of the arm allocation, to the extent possible.

### Intervention delivery

The PSE team informs the intervention delivery team about the infants randomized to the intervention arm. The intervention delivery teams conduct the first visit within 24 h of enrolment.

The intervention delivery visits are designed to accommodate the implementation of various components of intervention such as health, nutrition, and psychosocial in a single visit. On a particular visit, the focus is either on nutrition and health of the infant and mother or early child stimulation and responsive care and psychosocial care and support of the mother, with adjustments made as necessary. This intervention includes counselling on early and exclusive breastfeeding, expressing breast milk, kangaroo mother care, responsive feeding, micronutrient supplementation, hand washing, family planning, and immunization. The schedule includes 11 visits (days 1, 3, 7, 10, 11, 14, 17, 21, 22, 24, and 28) in the first 28 days, followed by visits every 2 weeks in months 2 and 3, and monthly visits in months 4, 5, and 6. In addition to providing counselling and support, the team also acknowledges and praises good practices.

During each visit, FAs ask about the well-being of both the infant and the mother. If the infant needs urgent care, the FA informs the supervisor and facilitates referral to the nearest health facility based on the family’s preference. The FA checks the mother’s understanding of the counselling messages and summarizes the key messages at the end of each visit.

If any problem with breastfeeding or complementary feeding is identified, a lactation counsellor or nutritionist intervenes to resolve the problem. If the problem is not resolved, the infants are referred to the pediatrician at the collaborating hospital.

The intervention delivery team FAs are measuring compliance with intervention during their home visits. This is done by both asking questions to the mother and observing empty food supplement packets or counting remaining tablets.

The pediatrician at the study clinic, located within the collaborating hospital, provides care for the infants in the intervention group and assesses infants with growth failure and associated illnesses. The lactation counsellor and nutritionist conduct home visits to assess breastfeeding and complementary feeding practices and counsel the mothers. Mothers of all infants with growth failure are assessed by a psychologist for any psychosocial problems that may pose hurdles in taking optimal care of the infant.

### Strategies to improve adherence to interventions {11c}

A system for electronic surveillance has been established to monitor infants who need extra care to ensure high adherence to intervention.

### Relevant concomitant care permitted or prohibited during the trial {11d}

Both groups of participants (intervention and control) have the freedom to utilize the standard care pathways, which include complimentary services from the government’s health system.

### Process evaluation and quality control

Two types of visits are conducted: observed visits and independent visits. Observed visits closely monitor worker activities, including family interactions, counselling quality, and procedure adherence. Coordinators perform monthly independent visits to ensure team adherence and accurate data collection. During pregnancy surveillance, activities involve observing rapport-building, survey-related messaging, assessing LMP, and consenting. During pregnancy follow-up, coordinators monitor the process of delivering counselling messages over the phone and handling inquiries from the participants. For screening and enrollment, criteria assessment, consenting, and anthropometric measurements are observed. Observation of outcome ascertainment focuses on the adequacy of anthropometric assessment methods. During intervention delivery, supervisors observe feeding sessions, breastfeeding practices, and child stimulation activities for the first 6 months, and complementary feeding practices from 7 to 12 months, with all details related to compliance documented at each visit.

Supplements are provided every 2 weeks, and at the time of delivery, the team asks questions on supplement consumption since the last visit and counts the remaining tablets to monitor compliance.

### Outcome ascertainment {18a}

Infants in both the intervention and control arms are visited at home by an independent outcome ascertainment team in pairs, at infant ages 1, 3, 6, 9, and 12 months, to measure weight, length, and head and mid upper arm circumference (MUAC); assess infant care practices; and document the prevalence of reported illnesses in the previous 2 weeks and care-seeking for illness and hospitalizations since the last visit. Additionally, the team measures the weight and MUAC of mothers at 2, 6, and 12 months postpartum and assesses postpartum depression among mothers using the Edinburgh Postnatal Depression Scale (EPDS), which has been validated for use in India [63].

Weight measurements are obtained using digital weighing scales (Seca model 354; California, USA) with an accuracy of up to 10 g, while infant length measurements are taken using infantometers (Seca model 417; California, USA) with a precision of 0.1 centimeters. Head and MUAC are taken using a measuring tape (model 212; Seca, California, USA) [64–66].

Information on household consumption and expenditures (monthly expenses on food, rent, health care, utilities, maintenance, fuel, reimbursement of loans, and helpers; education and health care expenses in the preceding three months; annual expenses on insurance, and clothes) will be collected at enrollment and 6 and 12 months of age. Neurodevelopmental assessment is done by trained and standardized psychologists. Cognitive, motor, language, and socio-emotional development will be assessed at 12 months of age using the Bayley Scales of Infant and Toddler Development III (BSID-III) [67]. Infant temperament will be assessed at 12 months of age using the Infant Temperament Scale [67, 68]. The child’s home environment will be assessed using the Home Observation of the Environment (HOME) questionnaire (Infant and Toddler version [69]) by the trained field team through physical home visits. Additionally, the Global Scales for Early Development (GSED) scale will be used at 6 and 12 months to assess child development across multiple domains, including cognitive, motor, and social-emotional development. Blood samples (~10 ml) will be collected at 12 months of age for micronutrient assays. The samples will be centrifuged, and serum and blood pellet stored at – 80 °C in the field office. The micronutrient concentrations will be measured in accredited laboratories.

Dietary assessment is done using: a food frequency questionnaire (FFQ) and 24-h dietary recall (subsample) at 9-month and 12-month outcome visits. Mothers of infants will be asked to provide information regarding their child’s food consumption in the previous 24-h duration. The collected data from these recalls will be entered into the DietCal software [70].

Ultrasounds for assessment of preterm birth are done at designated ultrasound centers. A trans-abdominal USG is scheduled between 9 and 13 weeks of gestation to estimate gestational age calculated by fetal crown length. If CRL is > 95 mm, femur length and head circumference are used to assess gestational age. All digital images are taken by trained radiologists according to intergrowth standards. Ten percent of all USG scans are randomly selected and sent to external reviewer for quality assurance.

Participants who discontinue the intervention will be treated as censored data and will be included in the analysis up to the point of discontinuation.

### Training and standardization

Staff are trained in the overall study objectives, strategies, and in their job responsibilities. Additionally, each team receives intensive training in their area of work along with training in Good Clinical Practice (GCP) guidelines.

Inter- and intra-observer standardization exercises for weight, length measurements, head circumference, and MUAC were conducted at the beginning of the study and will be repeated every 6 months. Weighing scales and infantometers are calibrated daily using standard weights and length measurement rods [56].

The psychologists undergo inter- and intra-observer standardization exercises for Bayley assessments. In addition, 10% assessments will be done by 2 psychologists. Agreement between the measurements assessed by the intra-cluster correlation coefficient (ICC) and by calculating Lin’s concordance correlation coefficient [71].

### Study oversight

Coordinators designated for each activity oversee the work of their teams. Weekly status reports are shared with the investigators. Periodic review meetings are held between the study teams, coordinators, and investigators.

The Centre for Intervention Science in Maternal and Child Health (CISMAC) is responsible for the oversight of the study. Technical staff from CISMAC interact with the investigators through monthly conference calls and twice-yearly site visits to review the study progress.

### Data management {#19}

A data management center is set up in the field office where real-time data is transferred to the server. Data is captured electronically on tablets and mobile phones and uploaded to an access-controlled cloud server. Range and logical checks have been incorporated to reduce errors. Additional logical and across-form checks are run twice weekly. Queries generated are given to study teams, and necessary corrections to the database are logged.

### Data safety monitoring committee (DSMC) {21a, 21b}

CISMAC has established a DSMC to oversee the study’s progress and evaluate the safety of the intervention. The members include an epidemiologist, a statistician, a pediatrician, and a social scientist. The committee reviews data on adverse events to supplements and deaths quarterly and meets twice a year. The committee suggested that trial-stopping rules will be dependent on the number of interim analyses that may be planned. For efficacy, interim analysis each of the two strata O’Brien Fleming’s rule will be followed. This rule is a group sequential method that sets increasingly stringent boundaries for statistical significance as the data accrue, thus controlling the overall type I error rate. For safety interim analysis in each of the two strata, Pocock’s rule will be followed. This rule uses a constant boundary for statistical significance, allowing for regular monitoring of the data without inflating the type I error rate. The first interim analysis will be conducted when 50% of enrolments have occurred in both strata. These stopping rules will ensure that the trial can be stopped early if there is clear evidence of benefit or harm, while also maintaining the integrity of the study results. The approach taken to these analyses will be rigorous and will adhere to the highest standards of statistical practice.

### Harms {#22}

This is a low-risk study, and serious adverse events are unlikely. All deaths of enrolled participants will be reported to the local ethics committee and the DSMC.

## Statistical analysis {#20a, 20b, 20c}

### Definitions

Gestational age at birth is determined by subtracting the date of the dating ultrasound from the date of birth and then adding the gestational age assessed during that dating procedure, which follows the INTERGROWTH-21 protocol [72, 73]. Preterm births are defined as births occurring at < 37 completed weeks of gestation. Birth weight centiles are calculated using the INTERGROWTH-21 standard using weight captured within 72 h of birth and gestational age at birth [1]. SGA is defined as birth weight < 10th centile using the INTERGROWTH-21 standard [1].

Weight-for-age, length-for-age, and weight-for-length *z* scores are calculated based on weight and length measured at 12 months (± 28 days).

Proportion stunted, underweight, and wasted will be defined as having length-for-age *z* score < − 2 SD, WAZ < − 2SD, and weight-for-length *z* score < − 2SD, respectively, using WHO standards [56]. The neurodevelopmental outcome will be the composite scores of cognitive, language, motor, and socio-emotional scales and scaled scores of receptive language, expressive language, fine motor, and gross motor domains of BSID-III at 12 months of age.

#### Comparability between the two arms

Summary values (means, centiles, proportions) for sociodemographic characteristics in both arms will be presented in the baseline table.

### Main effects

The primary outcome will be analyzed using an intention-to-treat (ITT) approach. All participants randomized to a treatment group will be included in the analysis, regardless of their adherence to interventions. However, missing data on the primary outcome at 12 months will not be imputed since the key assumption that data will be missing at random would probably not be valid. However, we will use imputation methods depending on the nature of missingness for the secondary outcomes. Some authors might call the above approach modified intention-to-treat analysis; however, there is no clear consensus on the use of this terminology as per the CONSORT guidelines. The statistical precision of the effects will be presented as 95% confidence intervals (Cis). The distributions of WAZ will be presented in Epanechnikov kernel density plots by study arms. To quantify any differences in distribution, the 5th, 10th, 15th, 20th, and… up to the 95th percentiles will be compared using quantile regression. The 95% CI of the difference between the percentile estimates for the two arms will be calculated using bootstrap resampling with 1000 replacements. The percentile differences and corresponding CIs will be visualized in forest plots.

For binary outcomes, we will use generalized linear models (GLMs) of the binomial family with a log-link and an identity-link function to calculate the relative risks and risk differences, respectively. For continuous outcomes, GLMs of the Gaussian family with an identity-link function will be used to calculate the difference in means between the groups.

The effect of the intervention package on infant mortality across both strata will be estimated in a Cox proportional hazards model taking the stratification variable into account. The days of follow-up for each infant will be calculated as the date of enrolment subtracted from the date of the last follow-up or death.

We will adjust for imbalances in baseline features if present.

### Weight and length growth trajectories between birth and 12 months

A linear mixed-effects regression model with an unstructured covariance matrix will be used to examine the effect of the intervention on weight and length velocity from birth to 6 months and 6 to 12 months [74]. The effect of intervention on secondary outcomes will be assessed using the same methods as for the primary outcomes.

### Pre-specified sub-group analysis

Sub-group analyses will be conducted by maternal height (< 150 cm and ≥ 150 cm), underweight (MUAC < 23 cm and BMI < 18.5 kg/m^2^), years of education (< 12 and ≥ 12 years), and the wealth quintile of the household (defined both using an asset index and total household expenditures). Relative measures of effect within each of these sub-groups will be estimated. An a priori analysis will also be done for types of preterm, i.e., very (gestational age 28–32 weeks) and moderate, i.e., (gestational age 32–34 weeks) preterm.

The method for the generation of the asset index will be similar to the method used by the Demographic and Health Survey Programme (DHS Program) [75]. Gross total household expenditures will be calculated. The latter indicator of socioeconomic position will be used for concentration curves, concentration index (with 95% CI) and differences in the concentration index (with 95% CI) using the *F*-test to explore, summarize, and draw inferences on the equity impact of the intervention package. Additionally, absolute and relative inequalities in ponderal and linear growth of preterm and term SGA infants at 12 months using the slope index of inequality (SII) and relative index of inequality (RII), respectively, will be estimated. Outcomes having higher inequity in the descriptive analysis will be explored further. Stata (StataCorp LLC, College Station, Texas) and standard user-written packages will be used for all analyses [76–79]. The SPIRIT (Standard Protocol Items: Recommendations for Interventional Trials) reporting guidelines were utilized in preparing the methodology of this study (see Additional file 6, SPIRIT checklist) [80].

## Discussion

This study envisions that promoting the growth and neurodevelopment of vulnerable infants will contribute to targets defined for health and well-being for all to be achieved by 2030 under SDG 3. Studies have examined the effects of individual interventions on growth and neurodevelopment in preterm or term SGA infants and have observed low to modest effect sizes. This will be the first study to measure the efficacy of a comprehensive package of interventions on the growth and neurodevelopment of preterm and term SGA infants.

In selecting health and nutrition interventions, attention has been given to notable risk factors for outcomes affecting poor growth in vulnerable infants, such as maternal nutrition, maternal depression during the postpartum period, and sub-optimal infant feeding and responsive childcare practices. The findings of the study will help understand the extent to which growth can be improved when a comprehensive set of nutritional and healthcare needs of the mother and the infant are addressed in the first year of life. If successful, the study may reveal what is potentially achievable in the growth of small babies. The findings from this study will advance our scientific understanding and may help design relevant programs in India and other similar low-middle-income settings.

There are strengths and unique features of this study. First, this study will estimate the efficacy of the intervention package in two strata, i.e., preterm and term SGA separately with adequate power. Second, most of the intervention will be delivered at home through trained study workers, and compliance with this intervention will be observed when possible. The intention is to deliver the intervention with high quality to maximize the internal validity of the study findings.

The study has some limitations. This is an individually randomized trial. The design, therefore, limits the conduct of community mobilization activities. Despite the growing evidence of the role of domestic violence among women, child neglect, and child abuse in influencing birth and child growth and neurodevelopmental outcomes, interventions directly targeting these issues were not included in the package. The study investigators felt that intervening on such sensitive issues could potentially create resistance or other problems among the families and in the study communities.

## Conclusion

The findings emerging from the study will provide useful insights on the maximum achievable improvements in the growth and neurodevelopment of preterm or term SGA infants from lower middle socio-economic settings. The insights of the trial will help in strengthening the already existing maternal and child health programs in India and other low-middle income settings.

### Trial status {13}

**Fig. 2:**
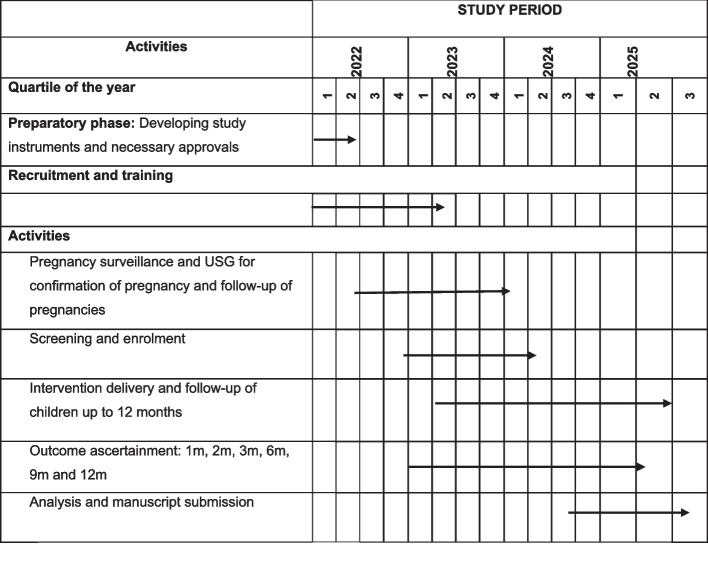
SPIRIT figure

Pregnancy surveillance by door-to-door survey started on 9th August 2022. Participant recruitment started on 24th January 2023 and is expected to be completed by 30th June 2024. The follow-up of the recruited infants will continue till 12 months of age [protocol version and date: Version 3.0, dated 24.07.2023; Fig. 2]. We are exploring additional funding to extend the intervention delivery till 24 months of age to estimate the efficacy of the intervention package on stunting, wasting, and neurodevelopment at 24 months of age.

### Supplementary Information

**Additional file 1.**

## Abbreviations

ASHA: Accredited Social Health Activist
BSID-III: Bayley Scales of Infant and Toddler Development, 3rd Edition
BMI: Body mass index
CI: Confidence interval
CISMAC: Centre for Intervention Science in Maternal and Child Health
CRL: Crown-rump length
DSMC: Data safety monitoring committee
ECD: Early child development
EPDS: Edinburgh Postnatal Depression Scale
FL: Femur length
GLM: Generalized linear model
HBNC: Home based neonatal care
HC: Head circumference
HMSC: Health Ministry’s Screening Committee
IFA: Iron-folic acid
ICMR: Indian Council of Medical Research
LMP: Last menstrual period
LAZ: Length-for-age *z* score
LBW: Low birth weight
MUAC: Mid-upper arm circumference
PSE: Pregnancy follow-up, screening and enrolment team
PST: Pregnancy surveillance team
RDA: Recommended daily allowance
SD: Standard deviation
SMD: Standardized mean difference
SGA: Small-for-gestation
USG: Ultrasonography
WAZ: Weight-for-age *z* score
WHO: World Health Organization
WLZ: Weight-for-length *z* score

## References

[1] Lee AC, Kozuki N, Cousens S, Stevens GA, Blencowe H, Silveira MF, Sania A, Rosen HE, Schmiegelow C, Adair LS (2017). Estimates of burden and consequences of infants born small for gestational age in low and middle income countries with INTERGROWTH-21^st^ standard: analysis of CHERG datasets. BMJ..

[2] Chawanpaiboon S, Vogel JP, Moller AB, Lumbiganon P, Petzold M, Hogan D, Landoulsi S, Jampathong N, Kongwattanakul K, Laopaiboon M (2019). Global, regional, and national estimates of levels of preterm birth in 2014: a systematic review and modelling analysis. Lancet Glob Health..

[3] Lawn JE, Ohuma EO, Bradley E, Idueta LS, Hazel E, Okwaraji YB, Erchick DJ, Yargawa J, Katz J, Lee ACC (2023). Small babies, big risks: global estimates of prevalence and mortality for vulnerable newborns to accelerate change and improve counting. Lancet..

[4] Blencowe H, Cousens S, Chou D (2013). Born too soon: the global epidemiology of 15 million preterm births. Reprod Health..

[5] Taneja S, Chowdhury R, Dhabhai N, Upadhyay RP, Mazumder S, Sharma S, Bhatia K, Chellani H, Dewan R, Mittal P (2022). Impact of a package of health, nutrition, psychosocial support, and WaSH interventions delivered during preconception, pregnancy, and early childhood periods on birth outcomes and on linear growth at 24 months of age: factorial, individually randomised controlled trial. BMJ..

[6] Lawn JE, Blencowe H, Oza S, You D, Lee AC, Waiswa P, Lalli M, Bhutta Z, Barros AJ, Christian P (2014). Every Newborn: progress, priorities, and potential beyond survival. Lancet..

[7] World Health Organization. International Classification of Diseases (ICD).In. Available from: https://www.who.int/standards/classifications/classification-of-diseases. Accessed 2023 Oct 10.

[8] Villar J, Cheikh Ismail L, Victora CG, Ohuma EO, Bertino E, Altman DG, Lambert A, Papageorghiou AT, Carvalho M, Jaffer YA (2014). International standards for newborn weight, length, and head circumference by gestational age and sex: the Newborn Cross-Sectional Study of the INTERGROWTH-21st Project. Lancet..

[9] Christian P, Lee SE, Donahue Angel M (2013). Risk of childhood undernutrition related to small-for-gestational age and preterm birth in low- and middle-income countries. Int J Epidemiol..

[10] Arcangeli T, Thilaganathan B, Hooper R, Khan KS, Bhide A (2012). Neurodevelopmental delay in small babies at term: a systematic review. Ultrasound Obstet Gynecol..

[11] Murray E, Fernandes M, Fazel M, Kennedy SH, Villar J, Stein A (2015). Differential effect of intrauterine growth restriction on childhood neurodevelopment: a systematic review. BJOG..

[12] Allotey J, Zamora J, Cheong-See F (2018). Cognitive, motor, behavioural and academic performances of children born preterm: a meta-analysis and systematic review involving 64 061 children. BJOG..

[13] Bos AF, Van Braeckel KN, Hitzert MM, Tanis JC, Roze E (2013). Development of fine motor skills in preterm infants. Dev Med Child Neurol..

[14] Klein VC, Rocha LC, Martinez FE, Putnam SP, Linhares MB (2013). Temperament and behavior problems in toddlers born preterm and very low birth weight. Span J Psychol..

[15] Cassiano RGM, Provenzi L, Linhares MBM, Gaspardo CM, Montirosso R (2020). Does preterm birth affect child temperament? A meta-analytic study. Infant Behav Dev..

[16] Pesonen AK, Räikkönen K, Strandberg TE, Järvenpää AL (2006). Do gestational age and weight for gestational age predict concordance in parental perceptions of infant temperament?. J Pediatr Psychol..

[17] Gorman KS, Lourie AE, Choudhury N (2001). Differential patterns of development: the interaction of birth weight, temperament, and maternal behavior. J Dev Behav Pediatr..

[18] Lin L, Amissah E, Gamble GD, Crowther CA, Harding JE (2020). Impact of macronutrient supplements on later growth of children born preterm or small for gestational age: a systematic review and meta-analysis of randomised and quasirandomised controlled trials. PLoS Med..

[19] Fenton TR, Groh-Wargo S, Gura K, Martin CR, Taylor SN, Griffin IJ (2021). Effect of enteral protein amount on growth and health outcomes in very-low-birth-weight preterm infants. J Acad Nutr Diet..

[20] Amissah EA, Brown J, Harding JE (2020). Protein supplementation of human milk for promoting growth in preterm infants. Cochrane Database Syst Rev..

[21] Mazumder S, Taneja S, Dube B (2019). Effect of community-initiated kangaroo mother care on survival of infants with low birthweight: a randomised controlled trial. Lancet..

[22] Lu LC, Lan SH, Hsieh YP, Lin LY, Chen JC, Lan SJ (2020). Massage therapy for weight gain in preterm neonates: a systematic review and meta-analysis of randomized controlled trials. Complement Ther Clin Pract..

[23] Conde-Agudelo A, Díaz-Rossello JL. Kangaroo mother care to reduce morbidity and mortality in low birthweight infants. Cochrane Database Syst Rev. 2016; 10.1002/14651858.CD002771.pub4. 10.1002/14651858.CD002771 11034759

[24] Bera A, Ghosh J, Singh AK, Hazra A, Mukherjee S, Mukherjee R (2014). Effect of kangaroo mother care on growth and development of low birthweight babies up to 12 months of age: a controlled clinical trial. Acta paediatr..

[25] Ohgi S, Fukuda M, Moriuchi H (2002). Comparison of kangaroo care and standard care: behavioral organization, development, and temperament in healthy, low-birth-weight infants through 1 year. J Perinatol..

[26] Feldman R, Eidelman AI (2003). Skin-to-skin contact (Kangaroo Care) accelerates autonomic and neurobehavioural maturation in preterm infants. Dev Psychol..

[27] Spittle A, Orton J, Anderson PJ, Boyd R, Doyle LW. Early developmental intervention programmes provided post hospital discharge to prevent motor and cognitive impairment in preterm infants. Cochrane datab system rev. 2015; 10.1002/14651858.cd005495.pub4. 10.1002/14651858.CD005495.pub4 PMC8612699 26597166

[28] Hamadani JD, Mehrin SF, Tofail F (2019). Integrating an early childhood development programme into Bangladeshi primary health-care services: an open-label, cluster-randomised controlled trial. Lancet Glob Health..

[29] Ferreira RC, Alves CRL, Guimarães MAP (2020). Effects of early interventions focused on the family in the development of children born preterm and/or at social risk: a meta-analysis. J Pediatr..

[30] Guerrant RL, Schorling JB, McAuliffe JF, de Souza MA (1992). Diarrhea as a cause and an effect of malnutrition: diarrhea prevents catch-up growth and malnutrition increases diarrhea frequency and duration. Am J Trop Med Hyg..

[31] Martorell R, Habicht JP, Yarbrough C (1975). Acute morbidity and physical growth in rural Guatemalan children. Am J Dis Child..

[32] Stephensen CB (1999). Burden of infection on growth failure. J Nutr..

[33] Manapurath RM, Gadapani Pathak B, Sinha B, et al. Enteral iron supplementation in preterm or low birth weight infants: a systematic review and meta-analysis. Pediatrics. 2022;150(Supplement 1) 10.1542/peds.2022-057092I 35921671

[34] Park JJH, Fang ML, Harari O (2019). Association of early interventions with birth outcomes and child linear growth in low-income and middle-income countries: Bayesian network meta-analyses of randomized clinical trials. JAMA Netw Open..

[35] Conde-Agudelo A, Díaz-Rossello JL. Kangaroo mother care to reduce morbidity and mortality in low birthweight infants. Cochrane datab system rev. 2016; 10.1002/14651858.cd002771. 10.1002/14651858.CD002771.pub4 PMC6464509 27552521

[36] Castanys-Muñoz E, Kennedy K (2017). Systematic review indicates postnatal growth in term infants born small-for-gestational-age being associated with later neurocognitive and metabolic outcomes. Acta paediatr..

[37] Zhang Z, Tran NT, Nguyen TS (2018). Impact of maternal nutritional supplementation in conjunction with a breastfeeding support program during the last trimester to 12 weeks postpartum on breastfeeding practices and child development at 30 months old. PLoS One..

[38] von Salmuth V, Brennan E, Kerac M, McGrath M, Frison S, Lelijveld N (2021). Maternal-focused interventions to improve infant growth and nutritional status in low-middle income countries: a systematic review of reviews. PLoS ONE..

[39] Cusick SE, Georgieff MK (2016). The role of nutrition in brain development. The Golden Opportunity of the "First 1000 Days". J Pediatr..

[40] Martorell R. Improved nutrition in the first 1000 days and adult human capital and health. Am J Hum Biol. 2017; 10.1002/ajhb.22952. 10.1002/ajhb.22952 PMC5761352 28117514

[41] Ong KK, Kennedy K, Castañeda-Gutiérrez E, *et al.* Postnatal growth in preterm infants and later health outcomes: a systematic review. Acta paediatr. 2015; 104(10):974-986. 10.1111/apa.13128 PMC5054880 26179961

[42] Varella MH, Moss WJ (2015). Early growth patterns are associated with intelligence quotient scores in children born small-for-gestational age. Early Hum Dev..

[43] Ruys CA, Hollanders JJ, Bröring T (2019). Early-life growth of preterm infants and its impact on neurodevelopment. Pediatr Res..

[44] Villar J, Giuliani F, et al. Monitoring the postnatal growth of preterm infants: a paradigm change. Pediatrics. 2018;141(2) 10.1542/peds.2017-2467 29301912

[45] Gladstone M, Oliver C, Van den Broek N (2015). Survival, morbidity, growth and developmental delay for babies born preterm in low and middle income countries - a systematic review of outcomes measured. PLoS One..

[46] Victora CG, Adair L, Fall C, et. al. Maternal and child undernutrition: consequences for adult health and human capital. Lancet. 2008; 371(9609):340-357. 10.1016/S0140-6736(07)61692-4 PMC2258311 18206223

[47] Puri VK (2008). VK Puri’s Handbook on Unauthorised Colonies & Constructions in Delhi: MCD/NDMC.

[48] Taneja S, Chowdhury R, Dhabhai N, Bahl R (2020). Impact of an integrated nutrition, health, water sanitation and hygiene, psychosocial care and support intervention package delivered during the pre- and peri-conception period and/or during pregnancy and early childhood on linear growth of infants in the first two years of life, birth outcomes and nutritional status of mothers: study protocol of a factorial, individually randomized controlled trial in India. Trials..

[49] Rogawski ET, Liu J, Platts-Mills JA, Kabir F, Lertsethtakarn P, Siguas M, Khan SS, Praharaj I, Murei A, Nshama R (2018). Use of quantitative molecular diagnostic methods to investigate the effect of enteropathogen infections on linear growth in children in low-resource settings: longitudinal analysis of results from the MAL-ED cohort study. Lancet Glob Health..

[50] World Health Organization. Managing possible serious bacterial infection in young infants when referral is not feasible. In. 2015. Available from: https://www.who.int/publications/i/item/9789241509268. Accessed 2023 Oct 10 26447263

[51] World Health Organization, UNICEF. Integrated management of neonatal and childhood illness. New Delhi: Ministry of Health & Family Welfare, Government of India.In. 2003. Available from: https://main.mohfw.gov.in/sites/default/files/7091371954. Accessed 2023 Oct 10.

[52] Hylander MA, Strobino DM, Dhanireddy R (1998). Human milk feedings and infection among very low birth weight infants. Pediatrics..

[53] Schanler RJ, Shulman RJ, Lau C (1999). Feeding strategies for premature infants: beneficial outcomes of feeding fortified human milk versus preterm formula. Pediatrics..

[54] World Health Organization. WHO recommendations for care of the preterm or low-birth-weight infant. In. 2022. Available from: WHO recommendations for care of the preterm or low-birth-weight infant. Accessed 2023 Oct 10. 10.1016/j.eclinm.2023.102155 PMC10518507 37753445

[55] Kuriyan R, Kurpad AV (2012). Complementary feeding patterns in India. Nutr Metab Cardiovasc Dis..

[56] WHO Multicentre Growth Reference Study Group (2006). WHO Child Growth Standards based on length/height, weight and age. Acta Paediatr Suppl..

[57] World Health Organization. Care for child development: improving the care of young children. In. Available from:https://www.who.int/publications/i/item/9789241548403. Accessed 2023 Oct 10.

[58] Squires J, Bricker D, Twombly E, Mounts L (2009). Ages & Stages Questionnaires: a parent-completed child monitoring system.

[59] ICMR-NIN Expert Group on Nutrient Requirement for Indians, Recommended Dietary Allowances (RDA) and Estimated Average Requirements (EAR).In: 2020. Available from brief_note.pdf (nin.res.in) Accessed 2023 Oct 10.

[60] World Health Organization. Thinking Health manual: an evidence-based approach to reducing prenatal depression. 2015. Available fromhttps://www.who.int/publications/ Accessed 2023 Oct 10.

[61] Ministry of Health and Family Welfare. Home Based Newborn Care Operational Guidelines (Revised 2014). New Delhi: Government of India; 2014. Available from: who.int/docs/default-source/operational-guidance/HBNBC. Accessed 2023 Oct 10.

[62] Johnson S, Moore T, Marlow N (2014). Using the Bayley-III to assess neurodevelopmental delay: which cut-off should be used?. Pediatric Res..

[63] Dubey C, Gupta N, Bhasin S, Muthal RA, Arora R (2012). Prevalence and associated risk factors for postpartum depression in women attending a tertiary hospital, Delhi, India. Int j soc psychiatry..

[64] SECA. SECA 417 - light and stable measuring board for mobile use. Hamburg: SECA. Available from: https://www.seca.com/en_us. 2013. Accessed 2023 Oct 10.

[65] SECA. SECA 354- digital baby scale. Hamburg: SECA. Available from: https://www.seca.com/en_us/details/seca354.html. 2013. Accessed 2023 Oct 10.

[66] SECA. SECA 212- measuring tape for head circumference. Hamburg: SECA. Available from https://www.seca.com/en_us/products/all-products/product-details/seca212.html

[67] Murray-Kolb LE, Rasmussen ZA, A et al. The MAL-ED cohort study: methods and lessons learned when assessing early child development and caregiving mediators in infants and young children in 8 low- and middle-income countries. Clin Infect Dis. 2014; 59 (Suppl 4):S261-S272. 10.1093/cid/ciu437 PMC4204608 25305296

[68] Taneja S, Sinha B, Upadhyay RP (2020). Community initiated kangaroo mother care and early child development in low birth weight infants in India-a randomized controlled trial. BMC pediat..

[69] Elardo R, Bradley RH (1981). The home observation for measurement of the environment (HOME) scale: a review of research. Develop Rev..

[70] DietCal. DietCal - a tool for dietary assessment and planning [Internet]. Available from: https://www.dietcal.com/ [Accessed 2023 Oct 10].

[71] Gulliford MC, Adams G, et. al. Intraclass correlation coefficient and outcome prevalence are associated in clustered binary data. J Clin Epidemiol. 2005;58(3):246-251 10.1016/j.jclinepi.2004.08.012. 10.1016/j.jclinepi.2004.08.012 15718113

[72] Ohuma EO, Papageorghiou AT, Villar J, Altman DG (2013). Estimation of gestational age in early pregnancy from crown-rump length when gestational age range is truncated: the case study of the INTERGROWTH-21st Project. BMC Med Res Methodol..

[73] Papageorghiou AT, Kemp B, Stones W, Ohuma EO, Kennedy SH, Purwar M, Salomon LJ, Altman DG, Noble JA, Bertino E (2016). Ultrasound-based gestational-age estimation in late pregnancy. Ultrasound Obstet Gynecol..

[74] Johnson W, Balakrishna N, Griffiths PL (2013). Modeling physical growth using mixed effects models. Am J Phys Anthropol..

[75] Rutstein SO (2015). Steps to constructing the new DHS Wealth Index.

[76] Araar A, Duclos JY (2007). DASP: Stata modules for distributive analysis.

[77] Bruun NH, Fenger-Gron M, Prior A: IC: Stata module to compute measures of interaction contrast (biological interaction). In.; 2017. Available from https://econpapers.repec.org/software/bocbocode/s457975.htm

[78] Jann B (2016). LORENZ: Stata module to estimate and display Lorenz curves and concentration curves.

[79] O’Donnell O, O’Neill S, Van Ourti T, Walsh B (2016). conindex: estimation of concentration indices. Stata J.

[80] Chan A-W, Tetzlaff JM (2013). SPIRIT 2013 explanation and elaboration: guidance for protocols of clinical trials. BMJ..

