# Supplementary material for: Impact of an integrated health, nutrition, and early child stimulation and responsive care intervention package delivered to preterm or term small for gestational age babies during infancy on growth and neurodevelopment: study protocol of an individually randomized controlled trial in India (Small Babies Trial)
Source: Trials. 2024 Feb 8;25:110. doi: 10.1186/s13063-024-07942-z (PMC10854034; doi:10.1186/s13063-024-07942-z)
Supplement: Supplementary file 1 — Additional file 1. [file 13063_2024_7942_MOESM1_ESM.docx]

**Additional file 1- Table: Details of intervention packages**

**Infants**

|  | **Intervention arm** | **Control arm** |
| --- | --- | --- |
| **Health and health services care seeking** | Identifying danger signs and seeking appropriate and early care for illness.  Appropriate management through study clinic and referral to the hospital if necessary  Prevention of infectious diseases through counseling and facilitation of timely immunization  Counselling and demonstration of correct handwashing practices to caregivers | Care routinely sought from usual sources-government (free of cost) and private providers.  Care routinely sought from usual sources-government (free of cost) and private providers. |
| **Nutrition**  **Exclusive Breastfeeding till 6 months of age**  **Kangaroo mother care during neonatal period**  **Micronutrient supplementation till 6 months of age**  **Optimal complementary feeding :6-12 months**  **Continued Breastfeeding till 12 months**  **Growth Monitoring** | Counselling and support for exclusive breastfeeding till 6 months of age.  Lactation counseling and support, including feeding of expressed breast milk if indicated (assessed by lactation counselor).  Promotion of and support to mothers/ caregivers for effective Kangaroo mother care  Daily vitamin D, Iron supplementation till 6 months as per WHO recommendations at 1 RDA dose. Micronutrients like vitamin A, Zinc and other B vitamins are given as multiple micronutrient supplementation based on their evidence.  Counselling and support for timely initiation, and continuation of complementary feeding  Demonstration of preparation of home-based foods and responsive feeding  Provision of milk cereal mix (125 kcal/ 5 g protein and 1 RDA micronutrients) as a supplement to breastfeeding to infants aged 6-12 months.  Counselling and support for continued breastfeeding till 12 months  Growth monitoring fortnightly in the first 2 months of life and subsequently monthly. Identification and management (medical and nutritional) of growth failure based on growth velocity and attained weight and length. |  |
| **Early Child play and stimulation** | Counselling, demonstration, and practice sessions for mothers and other family members on early child play and responsive care  Referral to a developmental pediatrician or psychologist if indicated. |  |
| **Home Visits for delivery of interventions and ensuring compliance with the above-mentioned interventions** | 11 visits in the first month: 6 visits (Day 1, 3, 7, 14, 21, 28) as in the national programme; 5 additional visits on days 10, 11, 17, 22, 24  Weekly in the 2^nd^ and 3^rd^ month  Fortnightly between 4^th^ to 12^th^ months |  |

**Mothers**

|  | **Intervention arm** | **Control arm** |
| --- | --- | --- |
| **Health** | Counselling on postnatal check-ups  Referral to the health facility, if necessary, for any illnesses  Counselling on family planning and provision of contraceptives if requested for | Care routinely sought from usual sources-government (free of cost) and private providers. |
| **Nutrition** | Multiple micronutrient supplementation    Provision of additional requirement of energy and protein through locally prepared snacks (600 Kcal energy and 20 g protein) in the first 6 months postpartum |  |
| **Psychosocial Support** | Promote positive thinking and problem-solving skills. |  |

**Additional file 2**. **Table. Nutritional composition of proposed milk cereal mix**

|  | **Composition of milk cereal mix** |
| --- | --- |
| **Nutrients** | **Per Sachet (In 25 g)** |
| Energy (kcal) | 126.9 |
| Protein (g) | 5.6 |
| Fat (g) | 5.5 |
| Vitamin A (mcg) | 400 |
| Vitamin D (mcg) | 5 |
| Vitamin E (mg) | 2.7 |
| Ascorbic Acid (mg) | 30 |
| Thiamine (mg) | 0.3 |
| Riboflavin (mg) | 0.4 |
| Niacin (mg) | 6.6 |
| Pantothenic acid (mg) | 1.8 |
| Pyridoxine (mg) | 0.3 |
| Vitamin B12 (mcg) | 0.7 |
| Zinc (mg) | 6.6 |
| Biotin (mcg) | 6 |
| Calcium (mg) | 100 |
| Iodine (mcg) | 90 |
| Potassium (mg) | 160 |
| Phosphorus (mg) | 90 |
| Magnesium (mg) | 54 |
| Selenium (mcg) | 10 |
| Copper (mcg) | 220 |
| Manganese (mg) | 0.06 |

**Additional file 3 Table. Composition of Riconia Silver**

| **Nutrients** | **RICONIA Silver (per tablet)** |
| --- | --- |
| Vitamin A (mcg/d) | 750 |
| Vitamin D (mcg/d) | 5 |
| Vitamin K (mcg/d) | 10 |
| Vitamin C (mg/d) | 50 |
| Vitamin E (mg a-TE/d) | 10 |
| Thiamine (mg/d) | 2 |
| Riboflavin (mg/d) | 3 |
| Niacin (mg/d) | 26 |
| Pyridoxin (mg/d) | 1.5 |
| Pantothenate (mg/d) | 5 |
| Folate (mcg/d) | 300 |
| Vitamin B12 (mcg/d) | 1 |
| Biotin (mcg/d) | 30 |
| Iron (mg/d) | 9 |
| Calcium (mg/d) | 162 |
| Zinc (mg/d) | 15 |
| Iodine (mcg/d) | 150 |
| Selenium (mcg/d) | 25 |
| Magnesium (mg/d) | 100 |
| Copper (mg/d) | 2 |
| Manganese (mg/d) | 2.5 |
| Phosphorus (mg/d) | 125 |
| Potassium (mg/d) | 40 |
| Phosphorus (mg/d) | 125 |
| Chloride (mg/d) | 36.3 |
| Chromium (mcg/d) | 25 |
| Molybdenum (mcg/d) | 25 |
| Nickel (mcg/d) | 5 |
| Silicon (mcg/d) | 10 |
| Vanadium (mcg/d) | 10 |

**Additional file 4- Table: Outcomes in children and timing of measurement**

| **Outcomes** | **Time points** | | | | |
| --- | --- | --- | --- | --- | --- |
|  | **Month 1** | **Month 3** | **Month 6** | **Month 9** | **Month 12** |
| Attained length, LAZ, WAZ |  |  | ✓ |  | ✓ |
| Attained weight, WAZ |  |  |  |  | ✓ |
| Stunted |  |  | ✓ |  | ✓ |
| Underweight |  |  | ✓ |  | ✓ |
| Wasted |  |  | ✓ |  | ✓ |
| Overweight or obesity |  |  |  |  | ✓ |
| Weight and length velocity (0 to 6, 6 to 12) | ✓ | ✓ | ✓ | ✓ | ✓ |
| Mid upper arm circumference |  |  | ✓ |  | ✓ |
| Head circumference |  |  | ✓ |  | ✓ |
| Hemoglobin in a sub sample |  |  |  |  | ✓ |
| Micronutrient status (Vitamin A, D, B12, Zinc, Iron, Folate, Selenium) in a sub sample |  |  |  |  | ✓ |
| Morbidity | ✓ | ✓ | ✓ | ✓ | ✓ |
| Hospitalizations | ✓ | ✓ | ✓ | ✓ | ✓ |
| Cognitive, language and motor scores, socio-emotional, temperament by Bayley Scales of Infant and Toddler Development (Bayley), Home environment by HOME Scale. |  |  |  |  | ✓ |
| GSED Neurodevelopment scale |  |  | ✓ |  | ✓ |
| Household consumption and expenditure  Cost of illness expenditure (care sought, treatment received, medical and transportation costs incurred)  Productivity losses (time lost to care-seeking, time lost to caretaking, travel time)  Payments (form of payment and origin of funding) |  |  | ✓ |  | ✓ |
| Dietary assessment (in a sub sample) |  |  |  | ✓ | ✓ |

**Additional file 5: Secondary outcomes in women and timing of measurement**

| **Outcomes (Postpartum)** | **Time points** | | |
| --- | --- | --- | --- |
|  | **Month 2** | **Month 6** | **Month 12** |
| BMI (weight; height measured at enrollment) | ✓ | ✓ | ✓ |
| Mid Upper Arm Circumference | ✓ | ✓ | ✓ |
| Depressive symptoms | ✓ | ✓ | ✓ |
| Dietary assessment (in a sub sample at 3 and 6 months) |  |  |  |

**Additional file 6 Table: WHO Trial Registry dataset items**

| No | Item |  |
| --- | --- | --- |
| 1 | Primary Registry and Trial Identifying Number | CTRI/2021/11/037881 |
| 2 | Date of Registration in Primary Registry | Registered on: 08/11/2021; Last modified on 28/09/2023 |
| 3 | Secondary Identifying Numbers | N/A |
| 4 | Source(s) of Monetary or Material Support | Centre for Intervention Science in Maternal and Child Health (CISMAC), Centre for International  Health, University of Bergen, Norway |
| 5 | Primary Sponsor | Dr Sunita Taneja |
| 6 | Secondary Sponsor(s) | N/A |
| 7 | Contact for Public Queries | Dr Ranadip Chowdhury |
| 8 | Contact for Scientific Queries | Dr Sunita Taneja |
| 9 | Public Title | Effect of Integrated Nutrition, Health, Care and Support Interventions delivered to small babies during infancy, on growth and neurodevelopment |
| 10 | Scientific Title | Impact of an integrated health, nutrition and early child stimulation and responsive care intervention package delivered to preterm or term small for gestational age babies during infancy on growth and neurodevelopment |
| 11 | Countries of Recruitment | India |
| 12 | Health Condition(s) or Problem(s) Studied | Either born preterm or term small for gestational age |
| 13 | Intervention(s) | **Infants**  **Health**  Facilitate medical care-seeking/access-  Counselling on Immunization  Counselling on handwashing practices  Provision of Zinc and ORS for the management of diarrhea  **Nutrition**  Counselling and support for Exclusive breastfeeding, expressed breastmilk feeding in first 6 months  Kangaroo mother care  Complementary feeding from 6 months  Micronutrient supplementation  Growth monitoring  Management of SAM  **Early Child Development** - Counselling and demonstration on early child play and responsive care (screening using ASQ III- 3, 6, 9 and 11 months)  **Mothers**  **Health**  Facilitate postnatal care  Counselling on contraception  **Nutrition**  Nutritional snacks in first 6 months (energy ~600 Kcal, proteins ~20g  Multiple micronutrients, iron-folic acid supplementation and calcium and Vit-D) tablets in first 6 months  **Psychosocial support**  Promote positive thinking and problem-solving skills in the first 12 months of child age  (screening with PHQ-2) |
| 14 | Key Inclusion and Exclusion Criteria | Inclusion: Preterm (< 37 weeks of gestation) or term SGA (birth weight for gestational age below the 10th percentile based on INTERGROWTH criteria) infants and their mothers. - Dating scan done within 20 weeks of gestation. - Caregiver consents to participate in the study.  Exclusion: Major congenital malformations  - Mother and baby intend to leave the study area within the next 12  months  -Baby or mother hospitalized after birth for more than 14 days |
| 15 | Study Type | Behavioral, Nutraceutical |
| 16 | Date of First Enrollment | 25/01/2023 |
| 17 | Target Sample Size | 2600 |
| 18 | Recruitment Status | Open to Recruitment |
| 19 | Primary Outcome(s) | Attained weight and Attained weight for age Z score at 12 months |
| 20 | Key Secondary Outcomes | Attained length, length for age, weight for length at 6 and 12 months of age  Attained weight, weight for age z-scores at 6 months of age  Attained Mid upper arm circumference (MUAC) and head circumference at 6 and 12 months of age  Proportion stunted, wasted and underweight at 6 and 12 months of age  Weight and length velocities between birth to 6 months, 6 to 12 months of age  Composite cognitive, language, motor, socio-emotional, temperament and HOME scores at 12 months of age  Global Scales for Early Development (GSED) at 6 and 12 months  Hemoglobin and micronutrient status (Vitamin A, D, B12, Zinc, Iron, Folate, Selenium) at 12 months of age in a sub-sample  Death during the first 6 and 12 months of age  Possible serious bacterial infections/severe infections in the neonatal period  Diarrhea and pneumonia in 0-5 months’ old and 6-12 months’ old infants  Hospitalization for illness from birth till 12 months of age  Household total expenditure at 6 and 12 months of age |
| 21 | Ethics Review | Yes |
| 22 | Completion Date | Ongoing |
| 23 | Summary Results | N/A |
| 24 | IPD Sharing Statement | We plan to share deidentified individual participant-level data (IPD) from our clinical trial. The shared IPD will include demographic information, baseline characteristics, and outcome data.  The data will be shared only after the main findings of the trial have been published. The exact timing will be determined based on the completion of the trial and the publication schedule. |

**Additional file 7- Table: Outcome measures**

| **Outcome** | **Specific measurement variable** | **Measurement tool** | **Analysis metric** | **Method of aggregation** | **Time point** |
| --- | --- | --- | --- | --- | --- |
| **Primary outcomes** | Attained weight (g) | Weighing scale | Weight at 12 months | Mean/ Median | 12 months of age |
|  | Weight-for-age Z-scores | Weighing scale and WHO growth chart | Weight at 12 months Z score at 12 months | Mean/ Median | 12 months of age |
|  |  |  |  |  |  |
| **Secondary outcomes**  **Infants** | Stunting | Infantometer and WHO growth chart | Binary variable indicating stunting (Z-score < -2) | Proportion | 6 months, 12 months |
|  | Wasting | Weighing scale, Infantometer and WHO growth chart | Binary variable indicating wasting (Z-score < -2) | Proportion | 6 months, 12 months |
|  | Underweight | Weighing scale and WHO growth chart | Binary variable indicating underweight (Z-score < -2) | Proportion | 6 months, 12 months |
|  | Overweight or obesity | Weighing scale, Infantometer and WHO growth chart | Binary variable indicating overweight (Z-score >+2) | Proportion | 12 months |
|  | Weight velocity (g/day) | Weighing scale and WHO growth chart | Change in weight over time | Rate of weight gain | Birth to 6 months, 6 to 12 months |
|  | Length velocity (cm/month) | Infantometer and WHO growth chart | Change in length over time | Rate of length gain | Birth to 6 months, 6 to 12 months |
|  | Neurodevelopment score | Bayley Scales of Infant and Toddler Development, 3rd Edition | Composite BSID-III scores for cognitive, motor, language and social-emotional domains  calculated using the raw scores and scaled scores | Mean/ Median | 12 months |
|  | Head circumference and Mid-upper arm circumference | Measuring tape | In cms on a continuous scale | Mean/ Median | 6 and 12 months |
|  | Developmental assessment | Global Scales for Early Development (GSED) | Developmental score (D-score) that captures multiple domains - cognitive, motor, language, and social-emotional development | Mean/ Median | 6 and 12 months |
|  | Morbidity and hospitalization | Caregiver reported (past two weeks) | Count at each assessment point | Proportion | 1,3,6,9,12 months of age |
|  | Dietary assessment – 24 hour dietary recall (Types and quantities of food consumed) | Standard Questionnaire for 24 hour dietary recall | Total calorie, protein and fat intake | Mean/ Median | 9 and 12 months (sub-sample) |
|  | Micronutrients (µg/dL) | Serum ELISA | Concentration of various micronutrients | Mean/ Median | 12 months (sub-sample) |
|  | Hemoglobin | Autoanalyzer | Hemoglobin level | Mean/ Median | 12 months (sub-sample) |
| **Maternal** |  |  |  |  |  |
|  | Depressive symptoms | Edinburgh Postnatal Depression Scale | Score on the Edinburgh Postnatal Depression Scale | Proportion | 2, 6, & 12 months |
|  | Mothers BMI and Mid-upper arm circumference (cm) | Weighing scale, Stadiometer  and Measuring tape | In cms on a continuous scale | Mean/ Median | 2, 6, & 12 months |
|  | Hemoglobin concentration (g/dL) | Autoanalyzer | Hemoglobin level | Mean/ Median | 12 months |
|  | Household consumption and expenditures (USD) | Questionnaire-based | Amount of household consumption and expenditures | Mean/ Median | 6 months, 12 months |
|  | Dietary assessment for mothers– 24 hour dietary recall  (Types and quantities of food consumed by mother) | Standard Questionnaire for 24 hour dietary recall | Total calorie, protein and fat intake | Mean/ Median | 3 months, 6 months |
